# Supplementary material for: (–)-Epicatechin Alters Reactive Oxygen and Nitrogen Species Production Independent of Mitochondrial Respiration in Human Vascular Endothelial Cells
Source: Oxid Med Cell Longev. 2022 Jan 11;2022:4413191. doi: 10.1155/2022/4413191 (PMC8767396; doi:10.1155/2022/4413191)
Supplement: Supplementary Materials — Supplementary data containing primer sequences, western blot images, and additional qPCR figures. Table S1: primer sequences for homo sapiens with product length. All primers were used under the same cycling conditions. Figure S1: stain-free blot images of individual western blot experiments. Repeat one (a), two (b), and three (c). Figure S2: western blot analysis of HUVEC lysates. (a) pThr172-AMPKα and total AMPKα, (b) pThr202/Tyr204-p44/42 MAPK and total p44/42 MAPK, (c) pSer1177-eNOS and total eNOS. Figure S3: gene expression responses following acute EPI treatment. HUVECs were treated with 0, 5, and 10 μM EPI over 48 h and lysed for analysis of gene expression. (a) Parkin, (b) PGC-1α, (c) Sirt1, (d) Tfam, (e) Catalase, (f) eNOS, and (g) NOX4. Data are means ± SEM from 3 independent experiments. Statistical significance was determined by a two-way ANOVA, with dose and time as factors. Multiple comparisons were performed by Dunnett's test to determine differences in gene expression between conditions. βMain effect of time (P < 0.05); ∗P < 0.05. [file 4413191.f1.zip › Table S1.docx]

| Gene | Accession | Sequence  Forward/Reverse or Anchor Nucleotide | Product length (bp) |
| --- | --- | --- | --- |
| RPL13a | NM_012423.4 | F: GGCTAAACAGGTACTGCTGGG  R: GGAAAGCCAGGTACTTCAACT | 104 |
| CAT | NM_001752 | AN: 1649 | 134 |
| SOD2 | NM_000636 | AN: 194 | 132 |
| DNM1L (DRP1) | NM_012062.5 | F: CACCCGGAGACCTCTCATTC  R: CCCCATTCTTCTGCTTCCAC | 99 |
| MFN2 | NM_014874.4 | F: CCCCCTTGTCTTTATGCTGATGT  R: TTTTGGGAGAGGTGTTGCTTATT | 168 |
| PPARGC1A  (PGC-1α) | NM_001330751.2 | F: TGCTAAACGACTCCGAGAA  R: TGCAAAGTTCCCTCTCTGCT | 67 |
| SIRT1 | NM_012238 | AN: 1382 | 109 |
| TFAM | NM_003201 | AN: 462 | 143 |
| NOS3 (eNOS) | NM_000603.5 | F: AACTATTTCCTGTCCCCGGC  R: AGGATTGTCGCCTTCACTCG | 173 |
| CYBB (NOX2) | NM_000397.4 | F: GGGCTGTTCAATGCTTGTGG  R: GGCCCATCAACCGCTATCTT | 80 |
| NOX4 | NM_016931.5 | F: CAGTCCTTCCGTTGGTTTGC  R: CAAAAGTTTCCACCGAGGACG | 189 |
| PRKN (PARKIN) | NM_004562 | AN: 747 | 91 |
| GABPA (NRF2) | NM_002040.4 | F: AAATTGAGATTGATGGAACAGAGAA  R: TATGGCCTGGCTTACACATTCA | 95 |
