# Supplementary figures and images for: (–)-Epicatechin Alters Reactive Oxygen and Nitrogen Species Production Independent of Mitochondrial Respiration in Human Vascular Endothelial Cells
Source: Oxid Med Cell Longev. 2022 Jan 11;2022:4413191. doi: 10.1155/2022/4413191 (PMC8767396; doi:10.1155/2022/4413191)

## Slide 1
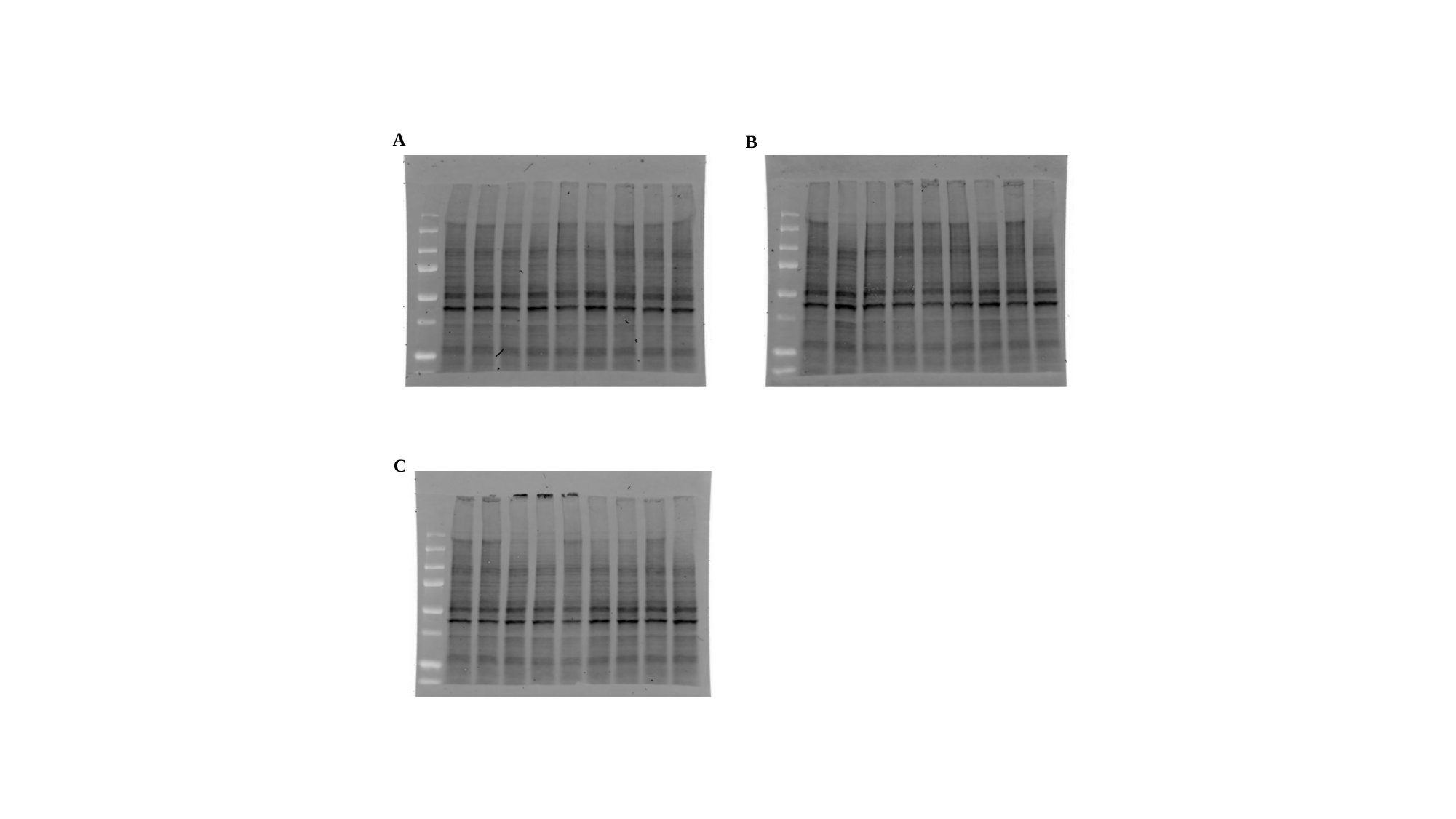

A
B
C

Supplement: Supplementary Materials — Supplementary data containing primer sequences, western blot images, and additional qPCR figures. Table S1: primer sequences for homo sapiens with product length. All primers were used under the same cycling conditions. Figure S1: stain-free blot images of individual western blot experiments. Repeat one (a), two (b), and three (c). Figure S2: western blot analysis of HUVEC lysates. (a) pThr172-AMPKα and total AMPKα, (b) pThr202/Tyr204-p44/42 MAPK and total p44/42 MAPK, (c) pSer1177-eNOS and total eNOS. Figure S3: gene expression responses following acute EPI treatment. HUVECs were treated with 0, 5, and 10 μM EPI over 48 h and lysed for analysis of gene expression. (a) Parkin, (b) PGC-1α, (c) Sirt1, (d) Tfam, (e) Catalase, (f) eNOS, and (g) NOX4. Data are means ± SEM from 3 independent experiments. Statistical significance was determined by a two-way ANOVA, with dose and time as factors. Multiple comparisons were performed by Dunnett's test to determine differences in gene expression between conditions. βMain effect of time (P < 0.05); ∗P < 0.05. [file 4413191.f1.zip › Fig S1.pptx]

## Slide 1
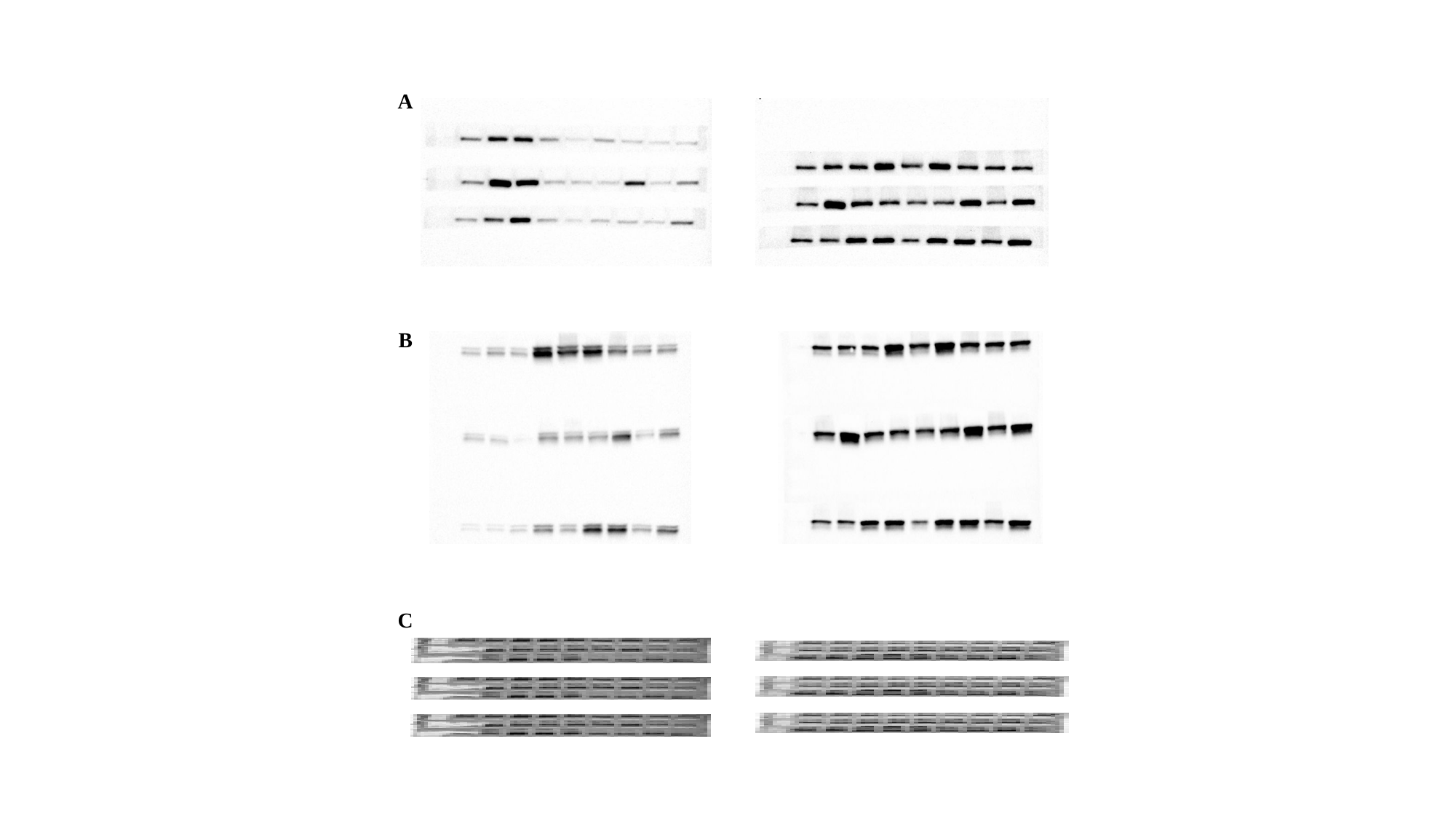

A
B
C

Supplement: Supplementary Materials — Supplementary data containing primer sequences, western blot images, and additional qPCR figures. Table S1: primer sequences for homo sapiens with product length. All primers were used under the same cycling conditions. Figure S1: stain-free blot images of individual western blot experiments. Repeat one (a), two (b), and three (c). Figure S2: western blot analysis of HUVEC lysates. (a) pThr172-AMPKα and total AMPKα, (b) pThr202/Tyr204-p44/42 MAPK and total p44/42 MAPK, (c) pSer1177-eNOS and total eNOS. Figure S3: gene expression responses following acute EPI treatment. HUVECs were treated with 0, 5, and 10 μM EPI over 48 h and lysed for analysis of gene expression. (a) Parkin, (b) PGC-1α, (c) Sirt1, (d) Tfam, (e) Catalase, (f) eNOS, and (g) NOX4. Data are means ± SEM from 3 independent experiments. Statistical significance was determined by a two-way ANOVA, with dose and time as factors. Multiple comparisons were performed by Dunnett's test to determine differences in gene expression between conditions. βMain effect of time (P < 0.05); ∗P < 0.05. [file 4413191.f1.zip › FigS2.pptx]
